# Impact of Blood Vessel Quantity and Vascular Expression of CD133 and ICAM-1 on Survival of Glioblastoma Patients

**DOI:** 10.1155/2017/5629563

**Published:** 2017-11-08

**Authors:** Ave Minajeva, Marju Kase, Mikk Saretok, Aidi Adamson-Raieste, Sandra Kase, Kristi Niinepuu, Markus Vardja, Toomas Asser, Jana Jaal

**Affiliations:** ^1^Faculty of Medicine, University of Tartu, Tartu, Estonia; ^2^East-Tallinn Central Hospital, Tallinn, Estonia; ^3^Haematology and Oncology Clinic, Department of Radiotherapy and Oncological Therapy, Tartu University Hospital, Tartu, Estonia; ^4^Riga Stradiņš University, Riga, Latvia; ^5^Neurology Clinic, Department of Neurosurgery, Tartu University Hospital, Tartu, Estonia

## Abstract

Glioblastoma (GB) is the most angiogenic tumor. Nevertheless, antiangiogenic therapy has not shown significant clinical efficacy. The aim of this study was to assess blood vessel characteristics on survival of GB patients. Surgically excised GB tissues were histologically examined for overall proportion of glomeruloid microvascular proliferation (MP) and the total number of blood vessels. Also, immunohistochemical vascular staining intensities of CD133 and ICAM-1 were determined. Vessel parameters were correlated with patients' overall survival. The survival time depended on the number of blood vessels (*p* = 0.03) but not on the proportion of MP. Median survival times for patients with low (<median) and high (≥median) number of blood vessels were 9.0 months (95% CI: 7.5–10.5) and 12.0 months (95% CI: 9.3–14.7). Also, median survival times for patients with low (<median) and high (≥median) vascular expression level of CD133 were 9.0 months (95% CI: 8.0–10.1) and 12.0 months (95% CI: 10.3–13.7). In contrast, the staining intensity of vascular ICAM-1 did not affect survival. In multivariate analysis, the number of blood vessels emerged as an independent predictor for longer overall survival (HR: 2.4, 95% CI: 1.2–5.0, *p* = 0.02). For success in antiangiogenic therapy, better understanding about tumor vasculature biology is needed.

## 1. Introduction

Glioblastoma (GB) is the most aggressive type of brain cancer in adults. Despite the use of multimodal treatment combinations, the prognosis of GB is still poor [1]. Since 1978, postoperative radiotherapy has been the mainstay of standard adjuvant treatment of GB [2]. However, in contrast to radiation-induced excellent local control rates in most solid tumors, nearly all GB patients die due to locally recurrent disease within 1 year after diagnosis [2, 3]. Additional chemotherapy with temozolomide, given concomitantly and after radiotherapy, has increased median survival time of GB patients only to 14.6 months [4], pointing towards the urgent demand for more effective treatment choices.

GB is one of the most angiogenic malignant tumors. Therefore, the inhibition of tumor angiogenesis has been an extremely attractive research area in neurooncology with a number of anticancer drugs (e.g., bevacizumab, cediranib, cilengitide, sunitinib, sorafenib, vandetanib, aflibercept, and tandutinib) being in various stages of clinical development for both newly diagnosed and recurrent GB [5–7]. Although these antiangiogenic therapeutics have shown high initial efficacy when used alone or in combination with standard treatments (including radiotherapy and chemotherapy), the duration of the tumor response is transient without a significant impact on patients' overall survival [5, 7–10]. The reasons for the lack of significant clinical efficacy of antiangiogenic drugs, however, are not fully elucidated [11], necessitating further look into the biology of GB vasculature.

Excessive and grossly disorganized blood vessel formation is a hallmark of GB. Next to typical intratumoral capillaries and bigger blood vessels, this tumor type contains also disease characteristic glomeruloid microvascular proliferation (MP). MP is defined as tangles of tiny vessels immersed in a complex mixture of irregularly ordered pericytes and extensive multilayered basement membrane. Although it is specific for glioblastoma, MP may also be found in a wide variety of human tumors, such as the stomach and breast cancer, where it is linked to unfavorable prognosis [12–14]. In anaplastic astrocytomas, these vascular proliferation types have been associated with rapid tumor growth and clinical progression. However, other studies have found no influence of MP on patients' survival with aggressive brain tumors, making clear conclusions about the significance of MP in glioblastoma difficult [15].

Blood vessels of GB can arise from sprouting and proliferation of endothelial cells from preexisting vascular networks (angiogenesis) and de novo through colonization of circulating bone marrow-derived endothelial progenitor cells that are recruited to the tumor (vasculogenesis). Also, extensive* in vitro* and* in vivo* studies have shown that, in GBs, a subpopulation of the multipotent CD133^+^ stem-like cell fraction is capable of differentiation into endothelial lineages and exerting alternative mechanisms of vascularization [16, 17]. Next to previously mentioned pathways, another mechanism in glioblastoma vascularization, tubular vasculogenic mimicry, has been described [18]. The latter term is used for “blood-conducting channels” that are nonendothelial cell lined and formed by tumor cells themselves. Consequently, blood vessels in GB may or may not contain endothelial cells and are very heterogeneous in their morphology and structure.

In addition to their distinct morphology, it is widely known that angiogenic blood vessels in tumors have abnormal function accompanied by high levels of local VEGF-A expression and increased expression of intercellular adhesion molecule-1 (ICAM-1) as well as permeability [19]. The roles of increased ICAM-1 expression and subsequent transendothelial migration of leucocytes have been reported to be controversial. Overexpression of ICAM-1 has been demonstrated to be involved in immune surveillance in breast, gastric, and colorectal cancers, but it was shown to promote tumor growth, progression, and angiogenesis in oral cancer [20]. In GB, the significance of increased ICAM expression is not fully elucidated.

The formation of vessels in GB and tumor blood supply involves vascular structures with various parameters whose influence on treatment efficacy and prognosis is not exactly known. Therefore, the aim of the present study was to assess various blood vessel characteristics on the survival of GBM patients. We assessed the prognostic significance of the quantitative amount of MP and all blood vessels as well as vascular expression of CD133 and ICAM-1. The latter mentioned markers were preferred over classical endothelial markers, since blood vessels in GB may not contain endothelial cells.

## 2. Materials and Methods

The present study was carried out with permission from the Research Ethics Committee of the University of Tartu.

Between January 2006 and December 2008, 42 patients with GB were operated on at Tartu University Hospital or North Estonian Medical Centre. All patients had maximal safe resection (none had biopsy only). After surgery, the patients were treated with postoperative three-dimensional radiotherapy (±chemotherapy). Characteristics of patients are listed in Table 1.

### 2.1. Treatment Planning and Treatment Parameters

Treatment planning was performed using CT/MRI scans and TPS XiO CMS treatment planning system. The gross tumor volume (GTV) encompassed the resection cavity and any residual tumor. A 2-3 cm margin was added to create clinical target volume (CTV). Critical tissues were spared (brainstem, chiasma). For planned target volume (PTV), a 0.5 cm margin was included. Treatments were performed using linear accelerators (30–60 Gy in 2.0 Gy fractions; group mean dose: 54 Gy). The prescribed dose was normalized to 100% at the isocenter and PTV was covered by 95% isodose surface (ICRU Report 50). None of the patients received concomitant and adjuvant chemotherapy with temozolomide (available in Estonia since 2010). However, for recurrent disease, 26 patients received chemotherapy with lomustine (CCNU).

### 2.2. Microscopy and Immunohistochemistry (IHC)

Surgically excised GB specimens were immediately fixed in the buffered 10% formalin (pH 7.4) for 24 hours and subsequently embedded into paraffin wax as routinely performed. From the resulting tissue blocks, serial 4 *μ*m paraffin sections were cut and placed on glass slides for standard hematoxylin-eosin (H&E) staining and immunohistochemistry (IHC). The diagnosis of GB was confirmed on the H&E stained slides by two independent pathologists.

The overall proportion of MP (based on the amount of MP per microscopic field and graded as low, medium, or high) was determined in H&E stained sections by an experienced pathologist. Studies on the morphology of angiogenesis have been based on H&E stained sections or immunohistochemical detection of microvessel markers, such as factor VIII, von Willebrand factor, CD34, CD31, and CD105 [21]. Since no single endothelial marker is perfect and both microvessel marker positive and negative vascular structures (e.g., vasculogenic mimicry) have been reported in GB [18, 22], we decided to determine the total number of blood vessels in H&E stained sections in high-power microscopic fields (×40) by counting vascular structures based on typical morphological appearance and the presence of the counted vascular lumen and intraluminal red blood cells. All visible blood vessels were counted in 6 randomly taken microscopic fields (not compromised by necrosis) and the mean number of vessels per microscopic field was calculated.

For immunostaining, solutions and buffers provided by Dako (Hamburg, Germany) were used. The sections were deparaffinized and incubated in the target retrieval solution (pH 9.0) in a 96°C thermostated water bath for 40 min and afterwards in a peroxidase blocking solution for 5 min at room temperature. Subsequently, the tissue sections were incubated with the specific anti-human CD133 (1 : 50; Biorbyt Ltd., #orb18124) or ICAM-1 (1 : 100, G-5; Santa Cruz Biotechnology; sc-8439, Lot #F2111) antibody at room temperature for 1 hour under humid conditions. After several washings, the antigen-antibody complex was visualized by using Dako REAL™ EnVision Detection System, Peroxidase/DAB+, Rabbit/Mouse. Slides were counterstained with hematoxyline, dehydrated, and coverslipped for light microscopy.

After GB immunostaining, vascular staining intensities of CD133 and ICAM-1 were determined in 5-6 randomly taken high-power microscopic fields (not compromised by necrosis; ×40) using an arbitrary score (0 = no staining, 1 = weak staining, 2 = moderate staining, and 3 = strong staining). The mean vascular staining intensity per microscopic field was calculated.

### 2.3. Statistical Analysis

The SPSS statistical software was used to calculate individual means, group means, and standard deviations of the mean as well as median values. Additionally, a Pearson correlation analysis was utilized. Based on the proportion of overall MP, patients were allocated into subgroups (low-medium and high). Also, according to the median values of all visible blood vessels, vascular staining intensities of CD133 and ICAM-1 patients were divided into subgroups <median (less than median) and ≥median (equal to and more than median). These subgroups were used in survival analysis. Overall survival (OS) was defined as the period from the date of operation to the date of death resulting from GB or to the date of the last analysis. Survival curves were created using the Kaplan-Meier method and differences between the groups were compared using the log-rank test. Multivariate analysis was performed using the Cox proportional hazards model. A *p* value < 0.05 was regarded as statistically significant.

## 3. Results

### 3.1. Proportion of MP and Total Number of Visible Blood Vessels in GB

The overall proportion of MP was determined by an experienced pathologist. Additional evaluation and scoring of slides (visible blood vessel numbers, staining intensities of CD133 and ICAM-1) were carried out in a blinded fashion by two independent researchers, whose results were in good accordance (*R* = 0.8, *p* < 0.001).

**Figure 1:**
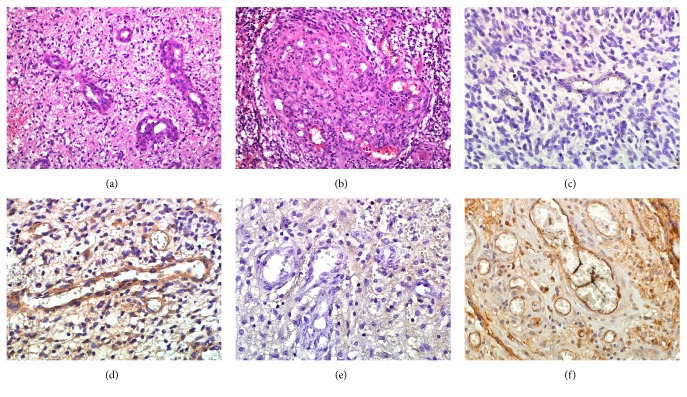
Blood vessels with various characteristics in glioblastoma (GB). The proportion of microvascular proliferation varied from low-medium to high. (a) Example of a case with sparsely located small microvascular proliferation (H&E, ×20). (b) Example of abundant large size microvascular tufts (H&E, ×20). (c) Immunohistochemical staining with anti-CD-133 antibody revealed portion of capillary blood vessels with CD133-negative staining (×40). (d) GB blood vessels with CD133-positive vascular staining (×40). (e) GB blood vessels with weak immunohistochemical staining for ICAM-1 (×40). (f) GB blood vessels with strong immunohistochemical staining for ICAM-1 (×40).

All GB sections contained MP, which is one of the hallmarks of this tumor type. The overall proportion of MP was low-medium (Figure 1(a)) in 39% and high (Figure 1(b)) in 61% of GB patients. Individual mean numbers of blood vessels were between 0.7 and 7.0 per microscopic field. The mean number of all visible blood vessels per high-power microscopic field in the whole study group was 2.5 ± 1.4 (mean ± SD) and the median number was 2.1.

### 3.2. Correlation of Vascular Staining Intensities of CD133 and ICAM-1 with the Total Number of Blood Vessels in GB

Positive staining intensity of CD133 was found in all types of GB blood vessels, including small capillaries and MP.  Figure 1 depicts CD133-negative (CD133−; Figure 1(c)) and CD133-positive (CD133+; Figure 1(d)) blood vessels in GB tissue. Individual mean values for CD133 staining intensity ranged from 0 to 1.8. In the whole group, the mean vascular CD133 staining intensity was 1.0 ± 0.5 (mean ± SD) and the median value was 1.0. A positive association was found between the number of visible blood vessels and vascular CD133 staining intensity (*p* = 0.03); that is, tumors in which stronger CD133 staining was detected contained more visible blood vessels.

Similar to the previous marker, a positive staining intensity of ICAM-1 (ICAM-1+) was found in all types of GB blood vessels.  Figure 1 illustrates GB blood vessels with weak (1E) and strong (1F) vascular staining intensity of ICAM-1. Individual mean values for ICAM-1 intensity were from 1.2 to 2.9. Group mean value was 1.8 ± 0.4 (mean ± SD) and the median value was 1.7. In contrast to CD133, between the number of visible blood vessels and vascular ICAM-1 staining intensity, a negative correlation was detected (*p* = 0.04); that is, in tumor samples with strong ICAM-1 staining, fewer blood vessels were seen.

### 3.3. Correlation of Blood Vessel Parameters with Overall Survival in GB Patients

**Figure 2:**
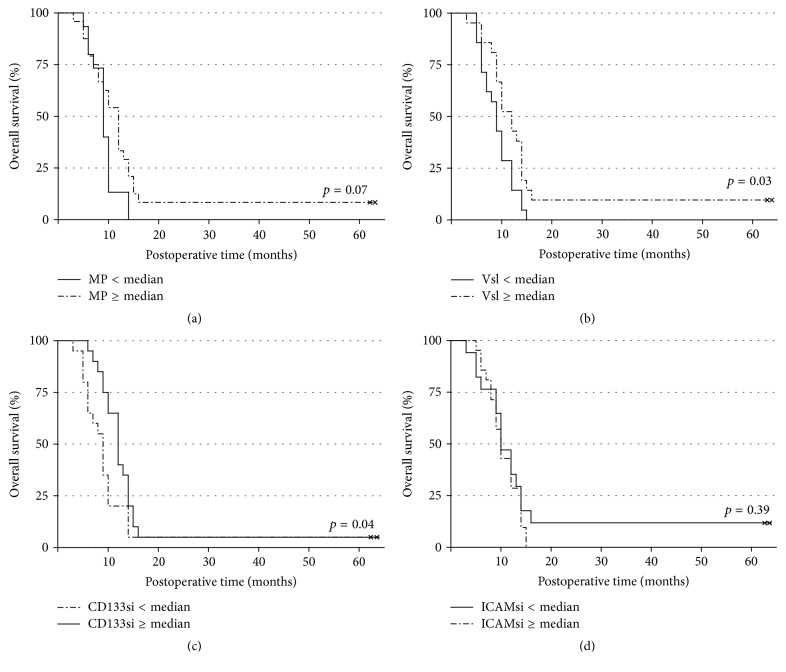
Kaplan-Meier analysis of overall survival (OS). (a) OS according to the proportion of glomeruloid microvascular proliferation (MP, low-medium versus high). (b) OS according to the number of all visible blood vessels (Vsl, <median versus ≥median). (c) OS according to the vascular staining intensity of CD133 (CD133si, <median versus ≥median). (d) OS according to the vascular staining intensity of ICAM-1 (ICAMsi, <median versus ≥median).

At the time of analysis, 40 patients out of 42 had died. The median OS of the whole study group was 10.0 months (95% CI: 9.0–11.0). The proportion of MP (low-medium versus high) did not significantly affect survival (log-rank test, *p* = 0.07), although a trend towards improved OS in patients with high proportions of MP in tumor tissue was evident (Figure 2(a)).

Figure 2(b) illustrates the OS among patients with low and high number of all visible blood vessels. The survival time clearly depended on the total number of visible blood vessels in GB tissue (*p* = 0.03). Median survival times for patients with low (<median) and high (≥median) number of blood vessels were 9.0 months (95% CI: 7.5–10.5) and 12.0 months (95% CI: 9.3–14.7), respectively.

Impact of vascular staining intensity of CD133 on survival of GB patients is depicted in Figure 2(c). The survival time of GB patients depended on the staining intensity of CD133 (*p* = 0.04). Median survival times for patients with low (<median) and high (≥median) immunohistochemical expression level of CD133 were 9.0 months (95% CI: 8.0–10.1) and 12.0 months (95% CI: 10.3–13.7), respectively. The staining intensity of vascular ICAM-1 did not affect survival (*p* = 0.39) of GB patients (Figure 2(d)).

In multivariate analysis (Table 2), the number of blood vessels (HR: 2.4, 95% CI: 1.2–5.0, *p* = 0.02) and Karnofsky Performance Score (HR: 3.0, 95% CI: 1.3–6.9, *p* = 0.01) emerged as significant independent prognostic factors for OS.

## 4. Discussion

GB is considered one of the most angiogenic tumors. The optimum protocol for antiangiogenic targeting in combination with radiation therapy and cytotoxic chemotherapy is, however, unclear and needs to be determined. For this, a better understanding of the biology of tumor vasculature is of paramount importance.

The present study showed that GB samples contain various types and amounts of blood vessels. In all tumor tissues, MP, as one of the characteristic features of GB, was seen. However, individual proportions of MP differed, being low-medium in 39% and high in 61% of GB patients. Additionally, the total number of all visible blood vessels varied between patients. In the study group, there were tumors that contained only 0.7 blood vessels per high-power microscopic field, whereas in some samples as many as 7.0 visible blood vessels were detected. Striking heterogeneity of the microvasculature in GB has also been reported in previous studies [6, 23, 24]. For example, it has been shown that GB may contain vascular rich areas with a plenty of small capillaries, areas with MP (especially around necrosis), and almost avascular fields, resulting in wide range (51.7–304.3 vessels/mm^2^) of vascular density values in 46 evaluated tumor cases [24].

GB tissue was seen to comprise CD133−, CD133+, and ICAM-1+ blood vessels. This is in good accordance with earlier studies where such types of blood vessels have also been seen [25–27]. In our study, a positive association was found between the number of all visible blood vessels and vascular CD133 staining intensity; that is, tumors in which stronger CD133 staining was detected contained also more blood vessels. It has been previously reported that CD133 potentiates proangiogenic activities of vascular endothelial growth factor (VEGF) and supports angiogenesis [28]. Moreover, knocking down CD133 in endothelial cells disrupts capillary formation* in vitro *and decreases angiogenesis* in vivo*, confirming a remarkable role of CD133 in angiogenesis [28].

Earlier studies have shown that CD133+ GB stem cells are able to form nonendothelial cell lined blood-conducting channels (“tubular vasculogenic mimicry”) [18, 22] but also transdifferentiate into endothelial cells [16, 25, 29]. Indeed, it has been reported that xenograft tumors derived from human CD133+ GB stem cells display widespread neoangiogenesis and significantly higher vascular density compared to CD133− cell formed tumors [30]. The participation of CD133+ GB stem cells in tumor blood vessel formation has also been confirmed in studies where these cells have been found to secrete markedly elevated levels of angiogenic growth factors such as VEGF, basic fibroblast growth factor, (bFGF), platelet derived growth factor (PDGF), and epidermal growth factor (EGF) [22, 30]. Also, gene expression analysis of 8 fresh, primary, and noncultured CD133+ GB cells showed upregulation of a number of genes (e.g., COL1A1, COL1A2, PGF, and TGFB1) involved in angiogenesis [31]. Importantly, in tumor tissue of 70 patients, a significant positive correlation has been found between the expression of CD133+ GB stem cells and CD133+ blood vessels [25]. Therefore, it is plausible that the majority of CD133+ blood vessels detected in the present study might have a tumor origin. The latter is also supported by the finding that a variable number (range: 20–90%, mean: 61%) of endothelial cells in GB carry the same genomic alteration as tumor cells, indicating that a significant portion of the vascular endothelium has a neoplastic origin [29]. In contrast to CD133, between the number of blood vessels and vascular ICAM-1 staining intensity, a negative correlation was detected; that is, in GB samples with strong vascular ICAM-1 staining, fewer blood vessels were seen. This suggests that, in the process of abundant angiogenesis, ICAM-1 might be downregulated. In fact, earlier* in vitro *studies have shown that the stimulation of normal and tumor-derived endothelial cells with angiogenic growth factors (VEGF, bFGF) results in substantially suppressed ICAM-1 expression [32]. Moreover,* in vivo*, significantly lower vascular density has been reported in the brain of ICAM-1 knockout mice after local infusion of VEGF [33].

Next to the morphological evaluation of tumor blood vessels, their impact on the survival of GB patients was determined. The median OS of the whole study group was 10.0 months, which is in good accordance with earlier studies where postoperative radiotherapy, as the main adjuvant therapy, was similarly utilized [2, 3]. The current study showed that the proportion of MP (low-medium versus high) did not significantly affect survival, although a trend towards improved OS in patients with high proportions of MP in tumor tissue was evident. This was somewhat surprising since MP is generally considered as one of the markers of the aggressiveness of the disease [34]. Morphological appearance of the MP is, however, often distorted and does not allow direct judgments concerning tumor blood flow in these blood vessels. Therefore, whether MP represents an accelerated form of effective angiogenesis or just a dysfunctional type of proliferation remains unclear. In spite of that, in a study of anaplastic oligodendroglial tumors that also included a proportion of patients with GB features, the presence of MP did not similarly influence survival [15]. In detail, GB patients with an oligodendroglial element (GBMO) had median survival time of 9 months and both stratified log-rank test (*p* = 0.6) and multivariate regression analysis (*p* = 0.1) showed no effect of MP on patients' overall survival.

When all visible tumor blood vessels were considered, a clear effect on survival of GB patients was detected. We found 3-month difference in survival, in favor of patients whose tumors contained higher number of visible blood vessels per high-power microscopic field. In addition, the multivariate analysis revealed that, next to the well-established prognostic factor KPS, the number of visible blood vessels emerged as the significant independent predictor for longer overall survival. Similar results have been published in a study of pediatric high grade gliomas mostly consisting of GB [35]. A significant relationship between the extent of vascular density (high versus low-medium) and survival was found in patients tending to survive longer if their tumor had higher density of CD31-positive vessels (HR = 0.9, *p* = 0.008).

It is well known that tumor oxygenation is an important determinant of the outcome of radiotherapy and possibly also of other treatment modalities in a number of tumor types [36]. In fact, it has been shown that tumor hypoxia induces both radioresistance and chemoresistance in glioblastoma preclinical* in vitro *and* in vivo *models [37, 38]. Therefore, better outcome of postoperative radiotherapy in more vascularized glioblastomas, presented in the current study, may be related to higher tumor perfusion and oxygen levels.

The present study revealed opposite effects of vascular CD133 and ICAM-1 expression on blood vessel numbers. Similar to the number of visible blood vessels, 3-month survival difference was detected in patients between low and high vascular expression levels of CD133 in favor of patients with stronger CD133 staining. In contrast, vascular ICAM-1 staining intensity did not affect survival. The latter has also been shown in GB study, where ICAM-1 gene expression did not correlate with overall survival of 14 patients. Moreover, distinct effects of blood vessels with different immunoprofiles on GB patients' survival have been reported in an earlier published study. It was shown that CD31-positive microvessel density (>median versus ≤median) did not affect survival of 29 GB patients, whereas CD105-positive microvessel density resulted in a significant difference in survival (mean survival times: 10.4 versus 18.6 months) [39]. Taken together, these data show that striking differences exist between vascular structures with various characteristics in GB tissue. Blood vessels differ by presence of endothelial cells, immunoprofile, effect on conventional treatment outcome, prognostic impact, and probably mechanisms by which the formation of vessels is stimulated or suppressed. Moreover, currently, it is not clear when and which type of blood vessel formation should be blocked. The latter might be one of the reasons why a vast majority of extensively tested antiangiogenic drugs have failed to show significant clinical efficacy.

The present study has several limitations. These include retrospective data collection and a small number of patients. Additionally, some important variables, such as tumor O6-methylguanine-DNA methyltransferase (MGMT) methylation status and recursive partitioning analysis (RPA), were not recorded.

## 5. Conclusions

The survival time of patients with GB depends on the number of blood vessels and vascular CD133 staining intensity prior to radiotherapy. For optimizing antiangiogenic therapy, a better understanding about tumor vasculature biology is clearly needed.

## Figures and Tables

**Table 1:** Characteristics of 42 patients with glioblastoma (GB).

Variable	No of patients (*n* = 42)	Percentage (%)
Gender		
(i) Male	23	55%
(ii) Female	19	45%
Age, years (range)^*∗*^	30–77	
Radiotherapy dose (range)	30–60 Gy	
Chemotherapy^*∗∗*^		
(i) No	16	38%
(ii) Yes	26	62%

^*∗*^Age at the time of operation; ^*∗∗*^Used for recurrent disease.

**Table 2:** Multivariate analysis for overall survival (OS).

Variable		OS	
		*p*	HR (95% CI)
No of visible blood vessels	<median vs ≥median	0.02	2.4 [1.2–5.0]
Radiotherapy dose^*∗*^	range 30–60 Gy	0.64	1.0 [0.9–1.1]
Chemotherapy	yes vs no	0.66	0.8 [0.4–1.8]
Karnofsky performance score	<70% vs ≥70%	0.01	3.0 [1.3–6.9]

^*∗*^Continuous variable; HR, hazard ratio; CI, confidence interval.

## References

[1] Crocetti E., Trama A., Stiller C. (2012). Epidemiology of glial and non-glial brain tumours in Europe. *European Journal of Cancer*.

[2] Walker M. D., Alexander E., Hunt W. E. (1978). Evaluation of BCNU and/or radiotherapy in the treatment of anaplastic gliomas. A cooperative clinical trial. *Journal of Neurosurgery*.

[3] Westphal M., Hilt D. C., Bortey E. (2003). A phase 3 trial of local chemotherapy with biodegradable carmustine (BCNU) wafers (Gliadel wafers) in patients with primary malignant glioma. *Neuro-Oncology*.

[4] Stupp R., Mason W. P., van den Bent M. J. (2005). Radiotherapy plus concomitant and adjuvant temozolomide for glioblastoma. *The New England Journal of Medicine*.

[5] Norden A. D., Drappatz J., Wen P. Y. (2008). Novel anti-angiogenic therapies for malignant gliomas. *The Lancet Neurology*.

[6] Lin C.-Y., Siow T. Y., Lin M.-H. (2013). Visualization of rodent brain tumor angiogenesis and effects of antiangiogenic treatment using 3D Δr2-*μ*MRA. *Angiogenesis*.

[7] Timotheadou E. (2011). New agents targeting angiogenesis in glioblastoma. *Chemotherapy Research and Practice*.

[8] Lai A., Tran A., Nghiemphu P. L. (2011). Phase II study of bevacizumab plus temozolomide during and after radiation therapy for patients with newly diagnosed glioblastoma multiforme. *Journal of Clinical Oncology*.

[9] Gilbert M. R., Dignam J. J., Armstrong T. S. (2014). A randomized trial of bevacizumab for newly diagnosed glioblastoma. *The New England Journal of Medicine*.

[10] Chinot O. L., Wick W., Mason W. (2014). Bevacizumab plus radiotherapy-temozolomide for newly diagnosed glioblastoma. *The New England Journal of Medicine*.

[11] Rahman R., Smith S., Rahman C., Grundy R. (2010). Antiangiogenic therapy and mechanisms of tumor resistance in malignant glioma. *Journal of Oncology*.

[12] Pettersson A., Nagy J. A., Brown L. F. (2000). Heterogeneity of the angiogenic response induced in different normal adult tissues by vascular permeability factor/vascular endothelial growth factor. *Laboratory Investigation*.

[13] Sundberg C., Nagy J. A., Brown L. F. (2001). Animal Model: Glomeruloid microvascular proliferation follows adenoviral vascular permeability factor/vascular endothelial growth factor-164 gene delivery. *The American Journal of Pathology*.

[14] Straume O., Chappuis P. O., Salvesen H. B. (2002). Prognostic importance of glomeruloid microvascular proliferation indicates an aggressive angiogenic phenotype in human cancers. *Cancer Research*.

[15] Smith S. F., Simpson J. M., Brewer J. A. (2006). The presence of necrosis and/or microvascular proliferation does not influence survival of patients with anaplastic oligodendroglial tumours: Review of 98 patients. *Journal of Neuro-Oncology*.

[16] Wang R., Chadalavada K., Wilshire J. (2010). Glioblastoma stem-like cells give rise to tumour endothelium. *Nature*.

[17] Soda Y., Marumoto T., Friedmann-Morvinski D. (2011). Transdifferentiation of glioblastoma cells into vascular endothelial cells. *Proceedings of the National Acadamy of Sciences of the United States of America*.

[18] El Hallani S., Boisselier B., Peglion F. (2010). A new alternative mechanism in glioblastoma vascularization: tubular vasculogenic mimicry. *Brain*.

[19] Nagy J. A., Chang S.-H., Dvorak A. M., Dvorak H. F. (2009). Why are tumour blood vessels abnormal and why is it important to know?. *British Journal of Cancer*.

[20] Usami Y., Ishida K., Sato S. (2013). Intercellular adhesion molecule-1 (ICAM-1) expression correlates with oral cancer progression and induces macrophage/cancer cell adhesion. *International Journal of Cancer*.

[21] Sharma S., Sharma M. C., Sarkar C. (2005). Morphology of angiogenesis in human cancer: a conceptual overview, histoprognostic perspective and significance of neoangiogenesis. *Histopathology*.

[22] Chen Y., Jing Z., Luo C. (2012). Vasculogenic mimicry-potential target for glioblastoma therapy: an in vitro and in vivo study. *Medical Oncology*.

[23] Wesseling P., Van der Laak J. A. W. M., De Leeuw H., Ruiter D. J., Burger P. C. (1994). Quantitative immunohistological analysis of the microvasculature in untreated human glioblastoma multiforme. Computer-assisted image analysis of whole-tumor sections. *Journal of Neurosurgery*.

[24] Izycka-Swieszewska E., Rzepko R., Borowska-Lehman J., Stempniewicz M., Sidorowicz M. (2003). Angiogenesis in glioblastoma - Analysis of intensity and relations to chosen clinical data. *Folia Neuropathologica*.

[25] He H., Niu C. S., Li M. W. (2011). Correlation between glioblastoma stem-like cells and tumor vascularization. *Oncology Reports*.

[26] Christensen K., Schroder H. D., Kristensen B. W. (2011). CD133^+^ niches and single cells in glioblastoma have different phenotypes. *Journal of Neuro-Oncology*.

[27] Lossinsky A. S., Mossakowski M. J., Pluta R., Wisniewski H. M. (1995). Intercellular Adhesion Molecule‐1 (ICAM‐1) Upregulation in Human Brain Tumors as an Expression of Increased Blood‐Brain Barrier Permeability. *Brain Pathology*.

[28] Adini A., Adini I., Ghosh K. (2013). The stem cell marker prominin-1/CD133 interacts with vascular endothelial growth factor and potentiates its action.. *Angiogenesis*.

[29] Ricci-Vitiani L., Pallini R., Biffoni M. (2010). Tumour vascularization via endothelial differentiation of glioblastoma stem-like cells. *Nature*.

[30] Bao S., Wu Q., Sathornsumetee S. (2006). Stem cell-like glioma cells promote tumor angiogenesis through vascular endothelial growth factor. *Cancer Research*.

[31] Garcia J. L., Perez-Caro M., Gomez-Moreta J. A. (2010). Molecular analysis of ex-vivo CD133+ GBM cells revealed a common invasive and angiogenic profile but different proliferative signatures among high grade gliomas. *BMC Cancer*.

[32] Griffioen A. W. (2008). Anti-angiogenesis: Making the tumor vulnerable to the immune system. *Cancer Immunology, Immunotherapy*.

[33] Kasselman L. J., Kintner J., Sideris A. (2007). Dexamethasone treatment and ICAM-1 deficiency impair VEGF-induced angiogenesis in adult brain. *Journal of Vascular Research*.

[34] Brat D. J., Van Meir E. G. (2001). Glomeruloid microvascular proliferation orchestrated by VPF/VEGF: A new world of angiogenesis research. *The American Journal of Pathology*.

[35] Smith S. J., Tilly H., Ward J. H. (2012). CD105 (Endoglin) exerts prognostic effects via its role in the microvascular niche of paediatric high grade glioma. *Acta Neuropathologica*.

[36] Bussink J., Kaanders J. H. A. M., Van Der Kogel A. J. (2003). Tumor hypoxia at the micro-regional level: Clinical relevance and predictive value of exogenous and endogenous hypoxic cell markers. *Radiotherapy & Oncology*.

[37] Hsieh C.-H., Wu C.-P., Lee H.-T., Liang J.-A., Yu C.-Y., Lin Y.-J. (2012). NADPH oxidase subunit 4 mediates cycling hypoxia-promoted radiation resistance in glioblastoma multiforme. *Free Radical Biology & Medicine*.

[38] Chou C.-W., Wang C.-C., Wu C.-P. (2012). Tumor cycling hypoxia induces chemoresistance in glioblastoma multiforme by upregulating the expression and function of ABCB1. *Neuro-Oncology*.

[39] Yao Y., Kubota T., Takeuchi H., Sato K. (2005). Prognostic significance of microvessel density determined by an anti-CD105/endoglin monoclonal antibody in astrocytic tumors: Comparison with an anti-CD31 monoclonal antibody. *Neuropathology*.

